# Reviewing the Impact of Social Media on the Mental Health of Adolescents and Young Adults

**DOI:** 10.7759/cureus.30143

**Published:** 2022-10-10

**Authors:** Chirag Gupta, Dr. Sangita Jogdand, Mayank Kumar

**Affiliations:** 1 Pharmacology, Jawaharlal Nehru Medical College, Datta Meghe Institute of Medical Sciences, Wardha, IND

**Keywords:** digital media, teenagers, youth, mental health, adolescent, social networking sites, social media

## Abstract

Adolescents now cannot imagine their lives without social media. Practitioners want to be able to assess risk, and social media may be a new factor to take into account. The impression of the link between social networks and intellectual health holds a strong emphasis on adolescent and professional perspectives, although there is little research that underlies these beliefs. Sexting, privacy concerns, cyberbullying, and negative impacts on education and mental health are dangers connected with social media use in this population. However, ethical social media use can increase opportunities for connection and communication, boosting one's self-esteem, promoting one's health, and getting access to crucial medical information. Despite rising evidence of the harmful impacts of social media on adolescent mental health, there is still a paucity of empirical research on how teenagers understand social media, notably as a body of wisdom, or how they can use the larger modern media discourses to voice an opinion. The youth use smartphones and other media in high numbers, which leads to chronic sleep deprivation, having a detrimental impact on cognitive ability, school performance, and socio-emotional functioning. Smartphone and social media use among teenagers are associated with an increase in mental distress, self-harming behaviours, and suicidality, according to evidence from numerous cross-sectional, longitudinal, and empirical studies. Clinicians can collaborate with young people and their families to mitigate the potential risks associated with social media and smartphone use by employing open, nonjudgmental, and developmentally appropriate strategies, such as education and practical problem-solving.

## Introduction and background

Teenagers now have unprecedented access to digital content via a variety of gadgets, including smartphones, tablets, laptops, desktop computers, and gaming systems. Today's media landscape is more expansive and diverse than ever before. Social media is a key component of this ecosystem. Social media, in its broadest meaning, refers to any digital application or software that allows users to engage in social interaction. Nearly half (46%) of US teenagers report using the internet "nearly continuously" in 2018, an increase from 24% in 2015 [1]. The widespread use of new media has produced a complicated world that young people, parents, medical professionals, and legislators must manage. While this media environment has created several fresh hazards and concerns for young people's mental health, it has also offered several unique advantages and opportunities [2]. Studies suggest that social media may influence teens to adopt unfavourable attitudes and behaviours [3]. Media formats, including digital platforms and interactive engagement, collectively referred to as social media, encompass media including email, text, blogs, message boards, dating applications, connection sites, games, and social networking sites [4]. Social networking platforms are created to facilitate online communication and information sharing, which has increased during the past 10 years. Youth utilize at least one of the following seven social networking sites at a rate of 97% among all teenagers between the ages of 13 and 17: adolescents spend most of their online time on YouTube (85%), Instagram (72%), Snapchat (69%), Facebook (51%), Twitter (32%), Tumblr (9%), or Reddit (7%) [5]. Recent research on the relationship between online communities and depression has found a common connection between the use of social networks by adolescents and depression, though there are some places where these findings are contradictory (like the relationship between screen time and mental health issues) and the quality of the evidence is not of great importance [6].

## Review

The use of social media and depression in teens are "generally correlated"; however, there have been inconsistent results in certain areas (such as the relationship between time spent on social media and mental health issues), and the quality of the data is generally low [7]. Using social media may increase the risk of self-harm, loneliness, and a decrease in empathy, based on particular studies. Other research either showed no harm or suggested that certain people may benefit from using social media [8]. Social media increasingly has taken a key place in young people's lives due to the rapid evolution of the technology landscape in recent years. Both huge new obstacles and fascinating new opportunities have been brought forth by social media. Research is starting to show how particular social media interactions may affect young people's mental health [9]. On social media, adolescents may communicate with others, publish, like, and share. These are generally considered to be active users. However, adolescents can also utilise social media passively by lurking and viewing solely the content of others. The binary distinction between active and passive usage does not reveal whether a certain behaviour is goal-directed or suggestive of procrastination [10]. For instance, procrastination may be characterised as conversing with others while delaying work on a more vital activity. Keeping up with friends' lives might be the purpose of seeing other people's content rather than participating with others. The social or nonsocial nature of the usage is a crucial distinction between different kinds. There are considerable hurdles in comprehending and measuring these many digital technology applications. Philosophically and empirically combining all digital acts into a single predictor of pleasure would always reduce accuracy [11]. 

Methods

The terms like "social media", "teenagers", "mental health", "digital media", "adolescents", and "social networking sites" were searched for in a database like "PubMed". Only results pertaining to the English language were shown. If there were more than one published report from a similar study, the latest one was used. Only review articles that also had original data were taken into account.

Is social media good or bad?

Conceptually, grouping the behaviours and use patterns under a single name disregards the reality that they serve different objectives and provide different results. When digital technology is viewed as a generic activity, its countless possible forms are disregarded. In light of this, it is essential to acknowledge that the effects of digital technology on teenagers' well-being are multifaceted [12]. This empirical uncertainty is exacerbated by the dearth of established measurements of technology usage. For the vast majority of work, self-reports of such are utilised. Self-reports have been shown to be inaccurate and of low validity due to their poor association with objective assessments of technology use [13]. The correlation between self-reported smartphone usage patterns and objectively documented usage is, at best, weak. Self-reports and objective measures yield the same results when comparing internet usage in general. In addition to losing precision by putting all types of technology usage under one behavioural category, this category's measurement also contributes to a loss of precision when taken as a whole. To achieve accuracy, we must examine the implications of diverse applications, ideally as assessed by science [14].

The outcomes of these studies have frequently been ambiguous, with many indicating that social media use has a minor but significant detrimental influence on mental health. A rising body of research tries to provide more in-depth understanding of the factors that influence adolescent development [15]. Since social media uses a variety of digital methods, it is difficult to sum up how it affects young people as a whole. In order to utilise and respond to social media in either adaptive or maladaptive ways, it is important to first understand personal characteristics that some kids may be more prone to exhibit. The precise social media practices or experiences that put teens in danger must also be identified. If we specifically survey US teenagers, 31% feel the impacts are primarily beneficial, 45% believe they are neither positive nor destructive, and 24% think they are unfavourable [16]. Teens who viewed social media as advantageous stated that it let them connect with friends, learn new things, and meet people with similar interests. According to those who believe the repercussions are serious, social media increases the likelihood of (i) bullying, (ii) neglecting face-to-face encounters, and (iii) acquiring erroneous assumptions about other people's lives. In addition, there is potential for avoiding depression and suicide by identifying symptoms utilising content. The relationship between offline and online risk has emerged as a recurrent theme in this field of study. The notion that the virtual age and its impacts are too complicated, fast-changing, or subtle for us to completely comprehend or successfully lead young people through is contested, challenging a typical message to parents. Helping youth with their online experiences and interactions is more likely to involve many of the same principles that drive healthy development and form the foundation of good parenting. If this is the case, it is excellent news for parents and guardians since it shows that existing evidence-based therapies and initiatives will continue to be effective in supporting teenagers in the digital age, regardless of any physical changes. Mediators of the association between social and adolescent depression and suicidality would be the final issue to examine (e.g., gender, age, and parental involvement) [17].

**Table 1:** Various Social Media Applications Available on the Internet - A General List

Type Of Social Media Apps	Examples
Social Networking Sites	Facebook, Instagram, Twitter, Snapchat
Messengers	Facebook Messenger, Whatsapp, Viber, iMessage
Media Sharing Apps	Whatsapp, Snapchat, Instagram, Youtube, Tiktok
Blogging Platforms	Wordpress, Wikipedia
Fitness & Lifestyle Apps	Fitbit
Discussion Forums	Reddit, Twitter

Risks of using social media in young adults

Peer experiences have a significant influence on the development and persistence of psychopathology in adolescents. In the realm of social media, peer relationships can be more frequent, intense, and quick. Previous research has highlighted a number of specific online peer interactions as possible mental health risk factors [17]. Cyber victimisation, or being the subject of online bullying, is frequently associated with increased rates of self-harm, suicidal thoughts, and other internalising and externalising problems. In addition, social media peer pressure, such as rejection from peers, online fights, and drama or conflict, may place young people in jeopardy. Online, where adolescents have access to a variety of their peers as well as potentially harmful content, peer influence processes may also be increased. If young people are exposed to social media content depicting dangerous behaviour, their likelihood of participating in such behaviour may increase (such as drinking and other drug usage). It may be easy to access internet resources that deal with self-harm and suicide, which might increase the risk of self-harm among at-risk youth [18]. According to a recent study, 14.8% of young people referred to mental hospitals because they posed a risk to others or themselves had accessed online resources that promoted suicide in the two weeks leading up to their admission [19]. They choose to display pics of themselves on social sites, which results in a constant stream of messages and images that are frequently meticulously edited to portray people in a positive manner. This puts an impact on certain young people, causing them to start comparing their accomplishments, aptitudes, or looks negatively. Studies have connected upper levels of social networking comparison to the depressive symptoms of adolescents [20].

Finally, it's critical to consider the issue of displacement, or what other vital activities are being replaced by time spent on social media, when assessing how technology use affects teenage mental health. It is common knowledge that young people's sleep patterns have a significant impact on their development and mental health. But earlier research has consistently connected using a mobile device before bed with lower sleep quality results, which include shorter sleep lengths, decreased sleep quality, and weariness during the day. Notably, 36% of teenagers say they wake up at least once throughout the night to check their gadgets, and 40% say they use a mobile device within five minutes of going to bed [21]. Therefore, the effect of social media on sleep quality continues to be a significant risk factor for later mental health issues in young people, making it a crucial subject for continued study. The majority of research on the relationship between social media use and depressive symptoms has focused on how often and problematically people use it [22]. The majority of the studies considered in this review revealed a positive and reciprocal association between social media use and depression and, occasionally, suicidality. It is yet unknown whether there is a connection between using the drug and depression or suicidality, and it is also uncertain how much adolescents' vulnerability and the substance's use features affect this connection. It is also unknown if other environmental elements, such as parental guidance and support or cultural disparities, have an effect on this link. Although it's possible that moderate use is associated with better self-regulation, it's unclear whether this is the result of intermediate users having innately better self-regulation [23].

Benefits of social media

Although the majority of the discussion about young people and new media has focused on possible problems, there are now more chances than ever to support adolescent mental health thanks to the distinctive characteristics of the social media ecosystem. Using social media may offer opportunities for humour and amusement, identity creation, and creative expression, among other advantages Teenagers now own more mobile devices than ever before, and they utilize social media at levels never before seen. Given how strongly young people are lured to digital devices and the affordances they provide, as well as their increased demand for novelty, social approval, and affinity, this may not come as a surprise. As teenagers spend more time interacting with digital technology, there is an urgent need to understand the ramifications of this usage and employ new technologies to benefit rather than harm adolescents' mental health and well-being. We should instead emphasise that digital technology is neither beneficial nor evil in and of itself in light of the current public debate [24]. Social connection is one of the most well-known advantages of social media, with 81% of students reporting that it increases their sense of connectedness to their peers. Teenagers frequently consider connecting with friends and family as the main advantage of social media, and prior research typically confirms that doing so increases people's well-being, using social media to boost acceptability or a sense of belonging [25]. The potential of digital media for boosting adolescent mental health extends beyond its regular usage by adolescents to encompass cutting-edge uses in screening, treatment, and prevention in the medical field [26]. Regarding screening, earlier research has shown the possible viability of looking through social media pages for indications of depression or drug misuse [19]. In general, more sophisticated machine learning techniques have been developed to recognise symptoms of mental illness, such as melancholy, post-traumatic stress disorder (PTSD), and suicidality, on social media [27]. The majority of existing research on adolescent media consumption relies on self-report measures. Because research has only been done once, it is impossible to establish definitive conclusions about whether media use precedes and predicts harmful impacts on mental health. Adults frequently point the finger at the media for the issues facing the younger generation [28].

We shouldn't solely attribute media panics to the novel and the unknown because they are cyclical. Because of technology, teenagers' time management, worldview, and interpersonal interactions have undergone remarkable and quick changes. Thanks to social media, there is unprecedented potential to raise awareness of mental health issues, and social media-based promoting health programmes have been evaluated for a variety of cognitive and behavioural health diseases. Young individuals with mental health concerns have intriguing therapy choices thanks to social media's immediate accessibility and wide possibilities, including the chance to reach hard-to-reach locations [29]. Youth-focused mental health mobile applications are acceptable, according to preliminary data, but additional study is required to determine their value and efficacy. Due to the increasing importance of digital media in young people's lives, they are now faced with new challenges and opportunities. According to an increasing body of studies, social media usage among adolescents may affect their mental health. But given how quickly the digital media ecosystem changes, additional study is required [30].

## Conclusions

Technology in the digital age does not "happen" to people instead, people use technology effectively. According to studies, utilising digital technology won't harm the average teen, but that doesn't mean there aren't circumstances with negative and serious consequences. In this study, we found that in-depth research on social media usage found a link to adolescent depression. Most research is cross-sectional; hence longitudinal studies are needed. Social media is entrenched in young people's social and personal lives. Professional organisations advise parents, educators, and institutions based on inadequate and inconsistent information about youth and digital technology. Policies limiting teens' access to new technologies can be futile if these tools are essential to stimulate social contact or develop digital and relational (digitally mediated) skills for emerging economies. In terms of health, reaching young people during crises and when help is needed is most important. Access to a variety of friendships and services may boost teen well-being.
